# Smoking and dietary inadequacy among Inuvialuit women of child bearing age in the Northwest Territories, Canada

**DOI:** 10.1186/1475-2891-12-27

**Published:** 2013-02-22

**Authors:** Fariba Kolahdooz, Nonsikelelo Mathe, Lalage A Katunga, Lindsay Beck, Tony Sheehy, Andre Corriveau, Sangita Sharma

**Affiliations:** 1 Aboriginal & Global Health Research Group, Department of Medicine, University of Alberta, 5-10 University Terrace, 8303 112 Street, Edmonton, Alberta T6G 2T4, Canada; 2 Department of Pharmacology & Toxicology, Brody School of Medicine, East Carolina University, Greenville, USA; 3 School of Food and Nutritional Sciences, University College Cork, Cork, Republic of Ireland; 4 Office of the Chief Public Health Officer, Department of Health and Social Services, Government of the Northwest Territories, Yellowknife, Canada

**Keywords:** Arctic, Childbearing age, Dietary adequacy, Inuvialuit, Smoking

## Abstract

**Objective:**

The prevalence of smoking in Aboriginal Canadians is higher than non-Aboriginal Canadians, a behavior that also tends to alter dietary patterns. Compared with the general Canadian population, maternal smoking rates are almost twice as high. The aim of this study was to compare dietary adequacy of Inuvialuit women of childbearing age comparing smokers versus non-smokers.

**Research methods & procedures:**

A cross-sectional study, where participants completed a culturally specific quantitative food frequency questionnaire. Non-parametric analysis was used to compare mean nutrient intake, dietary inadequacy and differences in nutrient density among smokers and non-smokers. Multiple logistic regression analyses were performed for key nutrients inadequacy and smoking status. Data was collected from three communities in the Beaufort Delta region of the Northwest Territories, Canada from randomly selected Inuvialuit women of childbearing age (19-44 years).

**Results:**

Of 92 participants, 75% reported being smokers. There were no significant differences in age, BMI, marital status, education, number of people in household working and/or number of self employed, and physical activity between smokers and non-smokers. Non-parametric analysis showed no differences in nutrient intake between smokers and non-smokers. Logistic regression however revealed there was a positive association between smoking and inadequacies of vitamin C (OR = 2.91, 95% CI, 1.17-5.25), iron (OR = 3.16, 95% CI, 1.27-5.90), and zinc (OR = 2.78, 95% CI, 1.12-4.94). A high percentage of women (>60%), regardless of smoking status, did not meet the dietary recommendations for fiber, vitamin D, E and potassium.

**Conclusions:**

This study provides evidence of inadequate dietary intake among Inuvialuit of childbearing age regardless of smoking behavior.

## Introduction

Smoking amongst women of reproductive age has been linked to possible infertility [1]. In western countries, including Canada, maternal smoking during pregnancy is a major cause of intrauterine under-nutrition, leading to low body weight and head circumference at birth [2]. Aboriginal women in Canada, including Inuit, Inuvialuit, First Nations, Dene and Métis, experience a greater prevalence of poor maternal health outcomes such as preterm birth, small-for-gestational-age, stillbirth, neonatal and post neonatal death compared with non-Aboriginal women [3-7]. Inuvialuit women of childbearing age, living in Arctic Canada, may be isolated in remote communities with limited access to healthcare, in particular prenatal care, which may exacerbate poor maternal health outcomes. Moreover, Inuvialuit have a high prevalence of smoking daily (61%) compared with the rest of Canada (17%) [8]. Cigarette smoking is a source of pro-oxidants that promote oxidative stress and contributes to endogenous generation of free radicals via many different mechanism including the activation of inflammatory cells [9]. Oxidative stress is a known mechanism that precipitates the development of chronic disease, such as atherosclerosis and carcinogenesis [9,10]. In one puff of a cigarette, the gas phase of the smoke exposes the smoker to greater than 1015 free radicals [11] and the relationship between antioxidant depletion and reduced antioxidant intake may predispose smokers to the premature development of tobacco related mortality and morbidity [12]. Therefore, it has been hypothesised that poor nutritional status may be more pronounced in certain individuals with unhealthy lifestyle behaviors such as smoking [13,14]. Smokers consume fewer food items rich in fiber, antioxidants and phytochemicals and tend to prefer a meat/alcohol dietary pattern compared with non-smokers [15-17]. A meta-analysis of 51 surveys conducted in 15 different countries comparing nutrient intakes of smokers and non-smokers showed that smokers’ dietary intakes differed substantially from those of non-smokers, with smokers consuming more fat, alcohol, energy, saturated fat, cholesterol and less vitamins C, E and beta-carotene [18]. Although these differences are not reported for all population groups [19], individuals who smoke might benefit to a greater extent from a fruit and vegetables rich diet [12]. The clustering of both poor diet and smoking can induce physiological changes, such as increased endothelial damage, oxidized low density lipoproteins and atherosclerosis that increases risk for development of chronic diseases. In addition to its direct effect on tissues, smoking can contribute to unbalanced nutrient profiles through a combination of altered taste preferences, metabolism and demand of certain nutrients such as folate, beta-carotene, selenium, calcium and vitamin C [13,18-22]. Intake of micronutrients such as folate and vitamin B12 in the diet of women of reproductive age are essential for the health of any potential offspring. Folate and vitamin B12 may induce epigenetic changes as they are important methyl donors during pregnancy [23]. Vitamin E has the potential to influence airway development via epigenetic mechanisms because it influences gene expression and epithelial cell signaling [24]. Understanding the dietary patterns of Inuvialuit women of childbearing age who smoke is paramount to designing nutritional interventions specific to this population. The aims of this study were to describe general prevalence of smoking and analyze dietary adequacy among Inuvialuit women of childbearing age who are smokers versus non-smokers in the Northwest Territories (NWT), Canada, and to evaluate the risk of dietary inadequacy among smokers.

## Methods

### Study design and setting

All data were collected at baseline for the Healthy Foods North (HFN) nutrition and lifestyle intervention. The setting, recruitment methods and data collection procedures have been described in detail elsewhere [25]. Briefly, homes were randomly selected in the three communities in the NWT, using local housing maps. Subjects were chosen to participate in the study provided they were women aged 19–44 years, had lived in the community for at least six months, and were the main food preparers and shoppers for the household. Pregnant and breastfeeding women were excluded due to their different nutritional requirements. The communities ranged in size from 400 to 3,500 people, with two food stores in the smaller communities and three food stores in the largest community. The stores’ food supplies are provided by air freight year round and by barge/sealift during a small window of time in the summer months when the ice melts. The largest community represents the regional administrative centre, while the smaller ones are comparatively more remote and activities, such as hunting and fishing, are more a part of daily life.

Trained staff collected dietary data using a culturally appropriate, validated quantitative food frequency questionnaire (QFFQ) developed specifically for the study population [26]. All participants were contacted by the staff for an interview. If a participant agreed to do the interview at that time, then it was conducted immediately. If they preferred to wait, then the interview was scheduled for another time. Participants were contacted up to seven times; if still unavailable, the interviewers moved on to the next household using the pre-planned map. If they agreed to take part in the study, participants were informed about the objectives of the study after which they were asked to sign a consent form prior to the start of the interview. An interviewer fluent in the local language or an interpreter was used for participants whose primary language was not English. Participants were asked to report the frequency of consumption over a 30-day period by choosing from eight categories, which ranged from ‘never’ to ‘two or more times per day’. Supplement information was collected as part of the QFFQ. Three-dimensional food models (NASCO Company, 901 Jamesville Ave, Fort Atkinson, Wisconsin 53538), household units (e.g. bowls, mugs, and spoons), standard units (e.g. teaspoon) and local food packages were carefully chosen with input from local communities to best estimate the weight per portion of foods and beverages consumed. Information on demographics, socioeconomic status, and heights and weights of participants were also collected. Data were examined for completeness by the project coordinator and if any set of data was incomplete the interviewer contacted the respondent to obtain the missing information. The response rate was between 69-93% depending on the community sampled. Participants were given a CAD $25 gift certificate for a local store to thank them for their time. All dietary data (including alcohol) from QFFQs were coded and analyzed using Nutribase Clinical Nutrition Manager version 9 (Cybersoft Inc., Phoenix, AZ, USA), a computerized dietary database.

Institutional Review Board approval was obtained from the Committee on Human Studies at the University of Hawaii and the Office of Human Research Ethics at the University of North Carolina at Chapel Hill. The Aurora Research Institute in Inuvik, NWT, licensed the study.

### Data analysis

Smokers were defined as those who responded “yes” to the question ‘Do you smoke cigarettes?’ and non-smokers those who answered “no”. Descriptive statistics included BMI (normal weight 20-24 · 9kg/m2, overweight 25-29 · 9kg/m2, and obese ≥30kg/m2) [27], smoking status (yes/no), marital status (single/married or common law), education level (none or some junior high school/ junior high school or high school completed/ college, trade school or university completed) and measures of socio-economic status such as “people in household working for pay; and people in household who are self-employed.

Average daily energy and nutrient intakes were compared with the appropriate Dietary Reference Intakes (DRI) for women aged 19–30 years and 31–44 years. Dietary adequacy was determined using the Estimated Average Requirements (EAR) according to specific age groups, Adequate Intake (AI) values were used for dietary fiber, vitamin B5, potassium and sodium [28]. A non-parametric Wilcoxon rank-sum test was performed to test for significant differences in mean intakes of nutrients between smokers and non-smokers. The association between smoking and inadequate intake of key nutrients (vitamins D and A, calcium, iron and zinc) was also determined using logistic regression analysis adjusted for age (continuous), sex, education (‘none’-some junior high school; ‘junior high school completed’- high school completed; ‘some college/trade school’-university completed), number of household items in working condition (≤7; 8–12; >12), percentage of people in household working (0 vs >0), and percentage of people in household on income support (0 vs >0) and energy intake (log transformed). Differences were considered statistically significant at *p* < 0 · 05. All statistical analyses were performed using SAS statistical software, version 9 · 2 (Cary, NC. SAS Institute Inc).

## Results

A total of 100 participants completed the QFFQ. Three did not respond to the question on smoking and were excluded from analysis. Based on recommended criteria [29], five subjects whose estimated caloric intakes were extremely high (>29288 kJ/d) were excluded, leaving a final group of 92 participants. Of the 92 women, 75% (n = 69) reported being smokers (Table 1). Smokers and non-smokers were not significantly different (p > 0 · 05) in terms of mean age and BMI. In addition there were no significant differences in marital status, education, number of people in household working for pay, number self employed and physical activity between the groups. Mean energy intake for all women exceeded the DRI of 7531 kJ (Table 2) and there were no significant differences in nutrient and energy intakes between smokers and non-smokers. Most women were below the EAR for some micronutrients with greater than 60% below recommendations for dietary fiber, potassium, vitamins D, and E. There were no significant differences between percentage of smokers and non-smokers below the DRI for all nutrients. Smokers had a higher nutrient intake density (per 4184kJ) for sugar (p < 0 · 05) compared with non-smokers (Table 3). Multivariate logistic regression analysis revealed no association between smoking and vitamins D, A, and calcium inadequacies, however there was a positive association between smoking and inadequacies of vitamin C (OR = 2.91, 95% CI 1.17-5.25), iron (OR = 3.16, 95% CI 1.27-5.90), and zinc (OR = 2.78, 95% CI 1.12-4.94) (Table 4). Twenty-one percent of participants reported using one or more nutrient supplements in the past 30 days, with multivitamins being the most common. Three percent indicated taking a vitamin D-containing supplement (data not shown).

**Table 1:** Characteristics of Inuvialuit women of childbearing age by smoking status

**Variables**	**Smokers (n =69)**		**Non-smokers (n = 23)**		***p-value***^‡^
	**Mean**	**SD**	**Mean**	**SD**	
**Age (years)**	34.7	6.8	33.9	7.5	***0.6***
BMI^*^	30.4	8.8	31.6	10.4	***0***.***6***
BMI^*^category^†^	**N**	**%**	**N**	**%**	
Normal weight (<25.0 kg/m^2^)	20	36.4	7	38.9	
Overweight (25–29.9 kg/m^2^)	10	18.2	4	22.2	
Obese (≥30.0 kg/m^2^)	25	45.5	7	38.9	***0***.***7***
**Marital status**					
Single	23	33.3	13	56.5	
Married or common law	45	66.7	9	43.5	***1.0***
**Education**					
None/some junior high school	19	27.5	2	8.7	
Junior HS or HS completed	31	44.9	12	52.2	
College, trade school or university	18	27.6	9	39.1	***0***.***4***
**Number of people with income in household**					
0	15	21.7	6	20.1	
1	30	43.5	7	30.4	
2	17	24.6	7	30.4	
3 or more	7	7.3	3	13.1	***0***.***9***
**Number of self employed people in household**					
0	53	79.8	19	82.6	
1	14	20.3	4	17.4	
2	2	2.9	0	0	***0***.***4***

HS, high school.

^‡^ Fisher exact test was used.

^*^Based on World Health Organization classification [27].

^†^Numbers and percentages might not add up to the total *n* as a result of missing responses.

**Table 2:** Mean energy and nutrient intake and dietary adequacy among Inuvialuit women of childbearing age: smokers versus non-smokers

**Nutrients**	**Smokers**		**Non-Smokers**			**Smokers**	**Non-smokers**		
	**Mean**	**SD**	**Mean**	**SD**	**P-value**	**% below AMDR**^†^		**P-value**	**AMDR**^†^
Energy (kJ)	15664.9	7723.7	14673.3	6962.2	0.66	21.7	15.9	0.50	7536
% energy carbohydrate	48.7	12.7	45.3	12.8	0.29	30.4	34.8	0.80	45-65
% energy from fat	27.1	6.8	28.2	7.6	0.35	17.4	17.4	1.00	20-35
% energy protein	15.3	5.9	15.5	5.5	0.72	15.9	21.7	0.50	10-35
	**Mean**	**SD**	**Mean**	**SD**	**P-value**	% below EAR^*^		**P-value**	EAR^*^
Carbohydrate (g)	436.0	218.0	384.0	237.0	0.17	-	-	-	-
Fat (g)	110.0	59.0	104.0	53.0	0.87	-	-	-	-
Protein (g)	140.0	80.0	130.0	67.0	0.78	-	-	-	-
Sugar (g)	237.0	130.0	202.0	186.0	0.08	-	-	-	-
Fibre (g)^‡^	22.7	14.7	22.4	10.9	0.84	65.2	69.6	0.80	25.0^‡^
Poly-unsaturated fat (g)	17.1	11.3	16.3	8.1	1.0	-	-	-	-
Omega 3 (g)	1.8	1.1	1.7	1.0	0.9	-	-	-	-
Omega 6 (g)	16.7	14.6	16.4	8.4	0.36	-	-	-	-
Cholesterol (mg)	460.0	237.0	426.0	242.0	0.4	-	-	-	ALAP^§^
Alcohol	67.1	128.3	75.5	145.0	0.79	-	-	-	-
% energy from alcohol	9.3	14.8	11.5	19.7	0.85	-	-	-	-
Vitamin BI (mg)	2.6	1.4	2.8	2.4	0.79	4.4	4.4	1.00	0.9
Vitamin B2 (mg)	4.4	2.1	4.4	3.1	0.35	69.0	23.0	0.44	0.9
Vitamin B3 (mg)	39.1	20.8	41.4	32.9	0.81	1.5	4.4	-	11.0
Vitamin B5 (mg)^‡^	10.8	5.4	9.2	4.8	0.24	21.7	7.3	0.11	53.0^‡^
Vitamin B6 (mg)	2.9	1.7	3.3	3.2	0.91	5.8	17.4	0.10	1.1
Vitamin B-12 (μg)	14.2	10.4	16.8	13.6	0.65	0	1.5	1.00	2.0
Folate (μg) ^**||**^	469.0	249.0	417.0	154.0	0.48	30.4	39.1	0.45	320.0
Vitamin C (mg)	261.0	214.0	219.0	279.0	0.15	10.1	17.4	0.45	60.0
Vitamin A (RAE) (μg) ^∞^	421.0	324.0	418.0	330.0	0.97	26.1	30.4	0.79	500.0
Vitamin D (μg) ^**|**^	275.0	259.0	232.0	183.0	0.34	76.8	73.9	0.78	10.0
Vitamin E (mg) ^¶^	3.2	6.6	3.2	2.6	0.13	97.1	100.0	1.00	12.0
Magnesium (g)^‡^	394.0	173.0	358.0	148.0	0.32	23.2	34.8	0.28	265.0^‡^
Potassium (g)^‡^	4.3	1.9	3.8	1.6	0.31	63.8	69.6	0.8	4.7^‡^
Sodium (mg)^‡^	5.12	2.9	5.06	2.9	0.92	4.4	4.4	1.00	1.5^‡^
Iron (mg)	26.4	16.2	23.0	12.3	0.54	7.3	4.4	1.00	8.1
Calcium (mg)	1442.4	921.6	1295.6	743.9	0.44	39.1	23.2	0.20	800.0
Selenium (μg)	177.0	107.0	146.0	74.1	0.31	4.4	0.0	0.57	45.0
Zinc (mg)	20.0	11.2	18.1	10.0	0.53	5.8	13.0	0.17	6.8

^†^Acceptable Macronutrient Distribution Ranges (AMDR) estimated amounts of energy needed to maintain energy balance for women aged ≥19 y.

^*^Adequacy was determined using the Estimated Average Requirements (EAR) levels for women aged 19–50 y.

^‡^Adequate Intake (AI) levels for women aged 19–50 y.

^§^As low as possible.

^**||**^Dietary Folate Equivalent.

^∞^Retinol Activity Equivalents.

^**|**^As cholecalciferol.

^¶^As α–tocopherol.

**Table 3:** Nutrient density per 1,000 kcal for selected nutrients among Inuvialuit women of childbearing age; smokers vs. non-smokers

**Nutrients**	**Smokers (n = 69)**			**Non-smokers (n = 23)**			***p value***
	**Median**	**Mean**	**SD**	**Median**	**Mean**	***SD***	
Carbohydrate (g)	122	124	32	113	122	32	*0.29*
Fat (g)	30.1	31	7.5	31.3	33.1	8.4	*0.35*
Protein (g)	38.3	37.4	14.8	38.6	35.8	13.8	*0.71*
Sugar (g)	67.6	63.9	29.5	55.7	49.7	26.7	***0.05***
Fiber (g)	6.2	5.9	2.7	7.3	6.6	3.4	*0.18*
Saturated fat (g)	10.2	10.1	2.9	10.5	11.5	3.1	*0.39*
Monounsaturated fat (g)	10.9	11.2	2.9	11.4	11.9	3.2	*0.38*
Polyunsaturated fat (g)	4.64	4.41	1.69	4.88	5.08	1.4	*0.27*
Omega-3 (g)	0.5	0.5	0.2	0.5	0.5	0.3	*0.87*
Omega-6 (g)	4.4	4.1	2.3	4.9	1.5	4.9	*0.08*
Cholesterol (mg)	132	131	55	124	117.6	45	*0.68*
Vitamin B1 (mg)	0.7	0.7	0.3	0.8	0.7	0.4	*0.32*
VitaminB2 (mg)	1.3	1.2	0.9	1.3	1.2	0.6	*0.98*
Vitamin B3 (mg)	11	10.3	3.9	0.8	0.7	0.4	*0.41*
Vitamin B5 (mg)	3.4	2.9	2.8	3	3	1.5	*0.47*
Vitamin B6 (mg)	0.1	0.8	0	0.9	0.9	0.4	*0.17*
Vitamin B12 (μg)	3.8	3.4	2.1	4.6	4.1	2.8	*0.4*
Folate (μg)^*^	137.1	134.5	67.9	138.9	140.3	60.9	*0.82*
Vitamin C (mg)	71	60.2	49.8	39.2	43.7	56.6	*0.16*
Vitamin A (RAE) (μg)^∞^	240	222	116	221	207	96	*0.7*
Vitamin D (μg)^†^	76.9	63.1	68	68.8	56.9	47	*0.49*
Vitamin E (mg)^‡^	1.4	1.3	0.6	1.4	1.4	0.5	*0.63*
Magnesium (g)	116.2	110.5	59.7	113.7	113	43.9	*0.81*
Potassium (mg)	1271	1200	808	1200	1257	475	*0.94*
Sodium (mg)	1417	1411	497	1558	1374	783	*0.87*
Iron (mg)	7.2	6.5	3	6.9	6.9	2.7	*0.96*
Calcium (mg)	404	351	169	381	383	126	*0.9*
Selenium (μg)	52.5	44.5	36.9	45.2	38.7	19.3	*0.6*
Zinc (mg)	5.6	5.3	2.3	5.3	5.2	1.8	*0.82*

^*^Dietary Folate Equivalent.

^∞^Retinol Activity Equivalents.

^†^As cholecalciferol in the absence of adequate exposure to sunlight.

^‡^As alpha–tocopherol.

**Table 4:** Odds ratios (95% CI) associated with smoking status and nutrients inadequacy among Inuvialuit women of childbearing age

**Smoking status**	**Nutrients inadequacy OR (95% CI)**					
	**Vitamin A**	**Vitamin D**	**Vitamin C**	**Calcium**	**Iron**	**Zinc**
Non-smokers	1.00	1.00	1.00	1.00	1.00	1.00
Smokers	1.41 (0.58-3.42)	1.00 (0.42-2.44)	2.91 (1.17-5.25)	2.36 (0.77-1.35)	3.16 (1.27-5.90)	2.78 (1.12-4.94)

† Logistic regression models were adjusted for age (continuous), sex, education (none-some junior high; junior high school completed- high school completed; some college/trade school-university completed, number of household items in working condition (≤7; 8–12; >12), people in household working, people in household on income support, and energy intake (log transformed).

## Discussion

The aim of this study was to describe the dietary patterns of Inuvialuit women of childbearing age who smoke compared with those who do not smoke. The results show that, overall, Inuvialuit women of childbearing age had a high prevalence of smoking, and most importantly, for all women regardless of smoking status, diets were characterized by high energy intake and inadequate micronutrient intake. There were no significant differences in energy and nutrient intakes and nutrient density for most nutrients between smokers and non-smokers.

Aboriginal populations are the fastest growing populations in Canada. Inuvialuit are Indigenous to the NWT and represent a young population with an average age of 22 years compared to 40 years for the rest of the non-Aboriginal Canadian population [8]. The high prevalence of smoking in this population is in line with that reported recently by Statistics Canada [8]. The prevalence rate of smoking was reported as 61% which is three times that for the rest of Canada (17%). It is widely accepted that smoking impacts on diet through altering of dietary patterns and impacts the absorption of certain micronutrients [14,18]. As such, smokers are assumed to have poorer diets than non-smokers. In this group, there were no significant differences in dietary adequacy between smokers and non-smokers. However, it is well established that smokers compared with non-smokers require a greater intake of nutrients compared with non-smokers. For example, people who smoke may need as much as 140 mg/day of vitamin C compared with 60 mg/day for non-smokers [30]. Thus women who smoke in this population are likely to face greater effects of dietary inadequacy than non-smokers.

Living in isolated communities in Northern Canada with limited resources, large geographic distances, varying language groups, and differing cultural beliefs and traditions all contribute to the complexity of providing adequate access to healthcare and affordable and nutritious foods [31,32]. The high prevalence of smoking and dietary inadequacy has been reported in other Indigenous populations of women of childbearing age. In rural communities in North Queensland, Aboriginal and Torres Strait Islander women had a high prevalence of obesity, poor dietary adequacy, and alcohol and tobacco use, which increased the rates of poor maternal health outcomes [33]. Traditionally, the Inuvialuit diet contained abundant sources of nutrient rich foods which contributed considerably to micronutrient status. The current nutrition transition occurring in this population is creating a shift from traditional foods to greater consumption of non-nutrient-dense store bought foods, which is occurring rapidly among Indigenous Canadians in Arctic communities [34-36]. This diet transition is associated with high energy intake and low intake of key micronutrients [25,37]. Indeed this explains the excessive energy intake among Inuvialuit women of childbearing age shown in this study. This pattern of dietary inadequacy has been previously reported for Inuvialuit in Arctic Canada [38,39].

The diets of all women were low in micronutrients and in particular vitamins D, E and potassium. For women of childbearing age the effect of poor nutrition follows both infant and mother for decades, and in particular induces fetal programming for increased risk of chronic diseases in later life for the infant [40]. Poor maternal nutritional status affects infant birth weight, increases risk of neural tube defects and causes cognitive delays and learning difficulties [41]. The teratogenic effects of cigarette smoking are well established [2,6,42] and include preterm birth, small-for-gestational-age, stillbirth, neonatal/post-neonatal death and cognitive and learning difficulties. It is also known that Aboriginal women experience a higher rate of these poor pregnancy outcomes compared with their non-Aboriginal counterparts [4,5,7]. In this population of women of childbearing age, the combined effect of high smoking prevalence and dietary inadequacy has major implications for future healthcare delivery in this population.

Dietary and lifestyle information on this unique population is limited and this study provides valuable information on diet and smoking behavior. However, this study is not without its limitations. Dietary information collected from the QFFQ may include over-reporting. However, the QFFQ was developed and validated specifically for this population and our previous results showed that when nutrient intakes were categorized into quartiles, the QFFQ and 24-h recalls indicated relative agreement for 77% for energy and macronutrients, 86% for total sugar and 72% for micronutrients [26]. Inuvialuit are a relatively small population and because of the high prevalence of smoking it was difficult to include a sizable group of non-smokers. Dietary adequacy among smokers and non-smokers could have been better analyzed by stratification of smokers according to number of cigarettes smoked. In addition, past smoking behavior was not accounted for.

## Conclusion

Results showed that there were no significant differences between smokers and non-smokers in dietary intake. Overall, women of childbearing age had high diet inadequacy. It is expected that those who smoke will have worse maternal health outcomes than non-smokers.

## Competing interests

The authors declare that they have no competing interests.

## Authors’ contributions

FK conducted the data analysis, intepretation of results, manuscript drafting, and finalizing manuscript. NM intepreted results and participated in manuscript drafting, LAK conducted a critical review and manuscript drafting, LB participated in data collection, TS and AC critical review, and SS conceptualization, review. All authors read and approved the final manuscript.
